# Whole Exome Sequencing in Children With Type 1 Diabetes Before Age 6 Years Reveals Insights Into Disease Heterogeneity

**DOI:** 10.1155/2024/3076895

**Published:** 2024-09-26

**Authors:** Andreia Fiúza Ribeiro, Ana Laura Fitas, Marcela Oliveira Pires, Paula Matoso, Dário Ligeiro, Daniel Sobral, Carlos Penha-Gonçalves, Jocelyne Demengeot, Íris Caramalho, Catarina Limbert

**Affiliations:** ^1^ Pediatric Endocrinology Unit Hospital de Dona Estefânia São José Local Health Unit, Lisbon, Portugal; ^2^ Pediatric Department Hospital Prof. Doutor Fernando Fonseca Amadora Sintra Local Health Unit, Amadora, Portugal; ^3^ Comprehensive Health Research Centre (CHRC) NOVA Medical School Universidade NOVA de Lisboa, Lisbon, Portugal; ^4^ Pediatric Department Hospital de São Francisco Xavier Lisboa Ocidental Local Health Unit, Lisbon, Portugal; ^5^ Instituto Gulbenkian de Ciência, Oeiras, Portugal; ^6^ Blood and Transplantation Center of Lisbon Instituto Português do Sangue e da Transplantação, Lisbon, Portugal; ^7^ Immunosurgery Unit Champalimaud Foundation, Lisbon, Portugal; ^8^ Faculty of Sciences University of Lisbon, Lisbon, Portugal

**Keywords:** *β*-cell dysfunction, early-onset, MODY, type 1 diabetes, whole exome sequencing

## Abstract

**Aims:** This study is aimed at comparing whole exome sequencing (WES) data with the clinical presentation in children with type 1 diabetes onset ≤ 5 years of age (EOT1D).

**Methods:** WES was performed in 99 unrelated children with EOT1D with subsequent analysis to identify potentially deleterious rare variants in MODY genes. High-resolution HLA class II haplotyping, SNP genotyping, and T1D-genetic risk score (T1D-GRS) were also evaluated.

**Results:** Eight of the ninety-nine EOT1D participants carried a potentially deleterious rare variant in a MODY gene. Rare variants affected five genes: *GCK* (*n* = 1), *HNF1B* (*n* = 2), *HNF4A* (*n* = 1), *PDX1* (*n* = 2), and *RFX6* (*n* = 2). At diagnosis, these children had a mean age of 3.0 years, a mean HbA1c of 10.5%, a detectable C-peptide in 5/8, and a positive islet autoantibody in 6/7. Children with MODY variants tend to exhibit a lower number of pancreatic autoantibodies and a lower fasting C-peptide compared to EOT1D without MODY rare variants. They also carried at least one high-risk DR3-DQ2 or DR4-DQ8 haplotype and exhibited a T1D-GRS similar to the other individuals in the EOT1D cohort, but higher than healthy controls.

**Conclusions:** WES found potentially deleterious rare variants in MODY genes in 8.1% of EOT1D, occurring in the context of a T1D genetic background. Such genetic variants may contribute to disease precipitation by a *β*-cell dysfunction mechanism. This supports the concept of different endotypes of T1D, and WES at T1D onset may be a prerequisite for the implementation of precision therapies in children with autoimmune diabetes.

## 1. Introduction

Type 1 diabetes (T1D) is a chronic and devastating disease affecting 1.52 million children, adolescents, and young adults worldwide [1]. The disease results from the immune-mediated destruction of pancreatic *β*-cells and leads to lifelong exogenous insulin dependence. T1D is a multifactorial disease with a strong genetic component. The major T1D susceptibility locus maps to the Human Leukocyte Antigen (HLA) class II genes at 6p21, accounting for up to 50% of genetic T1D risk [2]. The other half of the genetic risk is provided by more than 57 loci located outside of the HLA region, each alone conferring a small effect on disease risk [3].

The increased knowledge regarding the T1D genetic susceptibility landscape has allowed the elaboration of polygenic risk scores, known as T1D-genetic risk scores (T1D-GRS) that combine the information from HLA and non-HLA alleles to improve the accuracy of diagnosis, prediction, and prognosis. T1D-GRS have proved efficient in distinguishing T1D from type 2 diabetes (T2D) [4] and from monogenic diabetes [5, 6].

Although T1D is the most common form of diabetes in children, other subtypes of diabetes may occur. Monogenic diabetes is a heterogeneous group of diseases that includes maturity onset diabetes of the young (MODY), neonatal diabetes mellitus, genetic syndromes with diabetes, and insulin resistance [7–9]. Monogenic forms of diabetes result in functional pancreatic *β*-cell defects causing partial insulin deficiency and moderate to severe hyperglycemia in early life [7].

MODY is the most common form of monogenic diabetes [7, 10]. It is an autosomal dominant disease caused by a pathogenic variant in one of the fifteen confirmed genes [11, 12] and presents with hyperglycemia typically before age 25 [12]. Depending on the studied population, MODY diagnosis in pediatric diabetes populations is variable with a reported prevalence of 1.0%–6.5% [8, 10, 13]. Moreover, MODY cases are often (around 80%) misdiagnosed as T1D or T2D [7]. Nonetheless, it is also conceivable that diabetes subtypes may coexist and distinct disease mechanisms may overlap in some individuals [10, 12].

In recent years, increasing evidence revealed a central role of *β*-cell dysfunction in T1D, placing intracellular physiological or metabolic stress as a significant contributor to their vulnerability to immune attack [14]. *β*-cells are known to be highly susceptible to endoplasmic reticulum (ER) dysregulation due to their high protein production and secretory activity [15]. Moreover, they express low levels of antioxidant enzymes [16] and antiapoptotic factor BCL-2 [17], making them poorly equipped to survive the inflammatory milieu of the islets. It is therefore conceivable that gene variants expressed by *β*-cells associated with enhanced oxidative/ER stress and/or *β*-cell dysfunction may increase their fragility and susceptibility to apoptosis. Identifying the correct diabetes etiology is essential to provide individualized management, treatment, and prognosis [7–9].

We hypothesize that in children with early-onset T1D (EOT1D), the presence of genetic defects affecting *β*-cell function may contribute to earlier disease onset. This study is aimed at evaluating the presence of potentially deleterious rare variants in known MODY genes by whole exome sequencing (WES) in a selected cohort of unrelated EOT1D children (age at diagnosis ≤ 5 years old) and to investigate genotype-phenotype associations.

## 2. Methods

### 2.1. Participants and Ethics

A cross-sectional cohort study, including children with diabetes onset ≤ 5 years of age, was followed at the diabetes clinic in a pediatric tertiary center (*n* = 99; 96% of European ancestry). The study was approved by the local and national ethics committees (authorization number #318/2016). Informed consent was obtained from parents or legal guardians. The study was carried out in accordance with the Declaration of Helsinki. A control group for genetic risk analysis including healthy participants with no history of T1D or hyperglycemia (*n* = 171) was selected within a cohort representative of the Portuguese population (Prevadiab 2) [18].

The participants donated blood once.

### 2.2. WES

Genomic DNA was prepared from the blood. For WES (outsourced to Novogene, Cambridge, UK), library preparation used the Agilent SureSelect Human All Exon V6 platform and 150 bp paired-end sequencing reads (100× on target coverage) were obtained from an Illumina platform (PE150). Variant discovery was performed at Instituto Gulbenkian de Ciência (IGC) on cleaned sequencing reads. Briefly, alignment to the human reference genome GRCh37 used the Burrows-Wheeler Aligner; recalibration, local realignment, and variant calling used the Genome Analysis Toolkit; and variant annotation utilized ANNOVAR, SNPEff, and variant effect predictor. Retrieved variants obeyed the following criteria: high base quality in all reads, good mapping quality, and no strand bias. For heterozygous variants, allele fraction had in addition to be consistent with heterozygosity (~50% for each allele in case of biallelic loci). Genes reported as causal for MODY, for which compelling or reasonable genetic evidence exists were analyzed, namely, *ABCC8*, *CEL*, *GCK*, *HNF1A*, *HNF1B*, *HNF4A*, *INS*, *KCNJ11*, *NEUROD1*, *PDX1*, and *RFX6* [11].

### 2.3. Variant Pathogenicity

An *in silico* evaluation of pathogenicity was performed for rare missense variants using 9 computational prediction tools that included single and meta predictors (SIFT: https://sift.bii.a-star.edu.sg/www/Extended_SIFT_chr_coords_submit.html; PolyPhen-2: http://genetics.bwh.harvard.edu/pph2; MutationTaster: https://www.mutationtaster.org; CADD https://cadd.gs.washington.edu/snv; PredictSNP: https://loschmidt.chemi.muni.cz/predictsnp; M-CAP: http://bejerano.stanford.edu/mcap; REVEL: https://sites.google.com/site/revelgenomics; metaLR: retrieved from http://www.ensembl.org/info/docs/tools/vep/index.html; and I-Mutant 2.0: https://folding.biofold.org/i-mutant/i-mutant2.0.html). A rare variant was considered potentially pathogenic/deleterious if at least 7 of the 9 predictors classified the variant as damaging. For nonframeshift insertions, *in silico* evaluation of pathogenicity included the prediction tools SIFT-Indel (https://sift.bii.a-star.edu.sg/www/SIFT_indels2.html), MutationTaster, MutPred-Indel (http://mutpred2.mutdb.org/mutpredindel/), VEST-Indel (http://hg19.cravat.us/CRAVAT/), and CADD. An indel was considered potentially pathogenic/deleterious if at least 4 of the 5 predictors classified the variant as damaging.

### 2.4. HLA Class II Genotyping and T1D-GRS

HLA-DRB1 and DQB1/A1 genotypes for EOT1D participants and controls were assessed with a Luminex-based SSOP typing array and sequence-based typing (Sanger) for allelic resolution, according to the manufacturer's protocols (One Lambda LABType SSO, Thermo Fischer Scientific, MA, United States, and HLAssure, Texas Biogene, Inc., Melbourne, Australia, respectively). Haplotypes were reconstructed from allele data using Arlequin v3.5 software. Single nucleotide polymorphism (SNP) genotyping was performed at IGC, using the Sequenom iPLEX assay and detected in a Sequenom MassARRAY K2 platform.

The T1D-GRS was calculated using 24 non-HLA SNPs with a call rate above 95%, previously shown to be associated with T1D (Table S1), assuming each susceptibility allele has a log-additive effect on T1D risk, to which the weight (ln OR) provided by the HLA class II genotype was added (Table S2), according to the formula GRS = *Σ* (SNP risk allele (0, 1, 2) × ln OR + weight HLA II genotype)/*n* alleles as in Patel et al. [5]. The classification high or low T1D-GRS was defined as an individual score above or below the value of the 95th percentile of the control group (no T1D), respectively [19].

### 2.5. Diabetes Clinical and Laboratory Data

Clinical data at diabetes onset (age, presentation, glycated hemoglobin (HbA1c), and fasting C-peptide), diabetes treatment, T1D family history, and current data (pubertal stage, body mass index (BMI), and glycemic control) were collected retrospectively from medical files. Diabetic ketoacidosis was defined by hyperglycemia (blood glucose > 200 mg/dL), blood pH < 7.3 or serum bicarbonate < 18 mmol/L, and ketonemia ≥ 3 mmol/L, according to ISPAD guidelines [20]. Antibody status was determined at diagnosis. Autoimmune markers (immunoglobulin anti-GAD65, anti-IA-2, and anti-IAA) were measured by radioimmunoassay. The cutoffs for positivity were 0.9, 0.75, and 1.0 units/mL for GAD65, IA-2 and IAA, respectively. The detection limit of C-peptide was 0.1 ng/mL.

### 2.6. Statistical Analysis

Statistical analyses were performed using GraphPad Prism software version 10.1.0 for Mac (GraphPad Software, San Diego, CA, United States).

## 3. Results

The analysis of WES from 99 EOT1D children focused on identifying potentially deleterious rare variants in 11 MODY genes, namely, *ABCC8*, *CEL*, *GCK*, *HNF1A*, *HNF1B*, *HNF4A*, *INS*, *KCNJ11*, *NEUROD1*, *PDX1*, and *RFX6*, through a set of *in silico* analyses (see Methods). Eight of the ninety-nine children (8.1%) carried each one a potentially deleterious rare variant in a MODY gene, all in heterozygosity (Tables 1 and 2). Five out of eleven MODY genes were found affected, and these were *GCK*, *HNF1B*, *HNF4A*, *PDX1*, *and RFX6*. Together, these represented seven variants as two unrelated children carried the same variant in *RFX6*. Other rare variants were distributed, with children pairs carrying different variants in *PDX1*, or different variants in *HNF1B*, and single participants identified with an *HNF4A* or a *GCK* rare variant.

Six of the seven variants found were indexed in gnomAD (Genome Aggregation Database) v2.1.1 which provides a comprehensive collection of genetic variants, including both common and rare variants found in large scale sequencing projects of human exomes and genomes. In contrast, the nonframeshift insertion in *GCK* appears to be novel as it was not found in publicly available databases or after extensive literature review.

We next confronted our *in silico* prediction of deleteriousness to ClinVar, a publicly available database that gathers the clinical significance of single genetic variants in humans. Most identified rare variants were either not reported in ClinVar (variants in *GCK* and *HNF4A*) or classified as variants with conflicting interpretations of pathogenicity (variants in *HNF1B*, *RFX6*, and *PDX1* found in patient 1). Moreover, the *PDX1* missense variant, identified in patient 2, is classified as pathogenic (Table 2).

Clinical characteristics of the eight EOT1D children carrying rare variants in MODY genes are summarized in Table 3. Five of them are male, and only one has a first-degree relative with T1D. At diagnosis, the mean age was 3.0 years (1.0–5.0), 50% presented with diabetic ketoacidosis, and the mean HbA1c was 10.5% (8.3–13.0). Five children showed low but detectable C-peptide, and only one was negative for islet autoantibodies (Patient 5). Currently, the mean age is 14.9 years (11.0–18.0) and all are pubertal. One of them was diagnosed with obesity (BMI *z*-score ≥ 2 SD). All participants maintain insulin therapy, most of them with a continuous subcutaneous insulin infusion. Only Participants 4 and 6 use multiple daily injections. The mean total insulin daily dose (TIDD) is 0.8 IU/kg/day (0.4–1.1), and the mean HbA1c is 7.6% (6.8–8.6). Compared to children without MODY variants (Table 4), those with MODY variants tend to exhibit a lower number of islet autoantibodies (one third of children with MODY variants have two or more autoantibodies whereas two-thirds of children without MODY variants show the same characteristic; *p* = 0.1781, by Fisher's exact test) and a more pronounced decrease in *β*-cell mass at diagnosis, as indicated by the lower fasting C-peptide (0.14 ± 0.03 vs. 0.33 ± 0.06 ng/mL; *p* = 0.053, by Mann–Whitney test), in participants with and without MODY variants, respectively. The mean age (3.0 vs. 2.8 years; *p* = 0.706, by Mann–Whitney test) and the mean HbA1c at diagnosis (10.5% in both; *p* = 0.725, by Mann–Whitney test) were similar between the groups with and without MODY variants.

**Figure 1:**
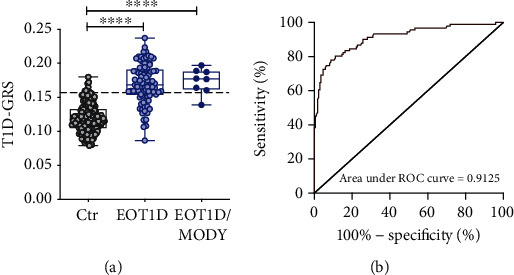
T1D-GRS is similar in EOT1D children regardless of the presence of rare variants in MODY genes. (a) Boxplot of T1D-GRS from 171 healthy controls (Ctr), 91 EOT1D children without deleterious rare variants in MODY genes, and 8 EOT1D children with potentially deleterious MODY rare variants. The central line within the box represents the median, and the upper and lower limits of the box represent the IQR (interquartile range). Lower and upper whiskers cover the lowest and highest 25% of data, respectively. The horizontal line shows a GRS equivalent to the 5th percentile for the control group, used to divide T1D children into high GRS (above the 5th percentile) and low GRS (below the 5th percentile). Mann–Whitney test was used for comparison between groups (^∗∗∗∗^*p* < 1 × 10^−4^). (b) ROC curve (area under the curve 0.9125) for T1D-GRS to discriminate EOT1D children without deleterious MODY rare variants (*n* = 91) from controls (*n* = 171).

All children with MODY variants carried at least one DR3-DQ2 or DR4-DQ8 T1D risk haplotype (Table S3). They had a T1D-GRS significantly higher than the control group and comparable to the score of EOT1D children not carrying these variants (Figure 1(a)). The T1D-GRS was highly efficient in discriminating between control individuals and EOT1D participants harboring MODY rare variants (area under ROC curve 0.9686, *p* < 0.0001) as well as between controls and EOT1D children without deleterious MODY rare variants (area under ROC curve 0.9125, *p* < 0.0001; Figure 1(b)). Notably, the T1D-GRS did not distinguish, within EOT1D children, individuals with or without MODY rare variants (area under ROC curve 0.5529), reflecting similar T1D genetic risk. Finally, the subdivision of the T1D-GRS into high and low based on the value of the 95th percentile of the control group [18] showed that 7/8 (87.5%) of EOT1D children carrying MODY rare variants had a high T1D-GRS (Table S3). Of note, the patient with a low T1D-GRS had nevertheless a score above the value of the 75th percentile of controls.

## 4. Discussion

This study demonstrates that potentially deleterious rare variants in MODY genes can be found in children with EOT1D, supporting the hypothesis that additional physiopathological mechanisms intrinsic to the *β*-cell may contribute to precipitate disease onset in the context of a high T1D genetic burden.

We found that 8 of the 99 unrelated EOT1D children (8.1%) enrolled in this study carry potentially deleterious heterozygous rare variants in MODY genes. This frequency is higher than the one reported for the general population (0.1%–1.5%) [10, 21], supporting that our EOT1D cohort is singular regarding potential *β*-cell disfunction. Potentially less anticipated, this frequency is also higher than those reported in other pediatric diabetes cohorts (1.0–6.5%) [8, 10, 13], a discrepancy that may well pertain to our inclusion criteria based on young age at diagnosis (≤ 5 years), independently of islet autoantibodies.

The identified rare variants were extensively scrutinized for their potential functional impact, and all passed our criteria. Our findings are strengthened when confronted with the literature. The *PDX1* variant found in patient 2 (p.E178K) has been demonstrated to increase PDX1 protein degradation and decrease its transcriptional activity [22]. Patient 1 carries the *PDX1* p.P33A variant, located in the transactivation domain of the protein in a highly conserved region. In a previous study, this variant has been classified as likely pathogenic and claimed to be associated with MODY [23]. Moreover, another variant in the same PDX1 amino acid position (p.P33T) was described as associated with MODY [24] and shown to lead to reduced binding to the insulin promoter and decreased transcriptional activity in vitro [25]. Some evidence also suggests that the rare variants found in *HNF1B*, in children 4 and 5, are damaging. The *HNF1B* variant found in patient 4 has been reported as clinically significant in two previous studies [26, 27], while the variant found in patient 5 was previously classified as pathogenic and associated with MODY [28, 29]. Even though we found no literature referring to the clinical significance of the *HNF4A* variant p.Y328C detected in patient 6, two variants in *HNF4A* located in adjacent amino acid residues (amino acids 326 and 327) have been associated with MODY [30]. While we expect the MODY mutations we uncovered to have functional impact, we do not anticipate they are causal for MODY but instead behave as mild hypomorphs.

In our cohort, several observations suggest that EOT1D children carrying deleterious rare variants in MODY genes do not represent *bona fide* monogenic diabetes children. We observed that 4/8 of these children present with ketoacidosis at diagnosis, a clinical feature absents in MODY [12]. Moreover, in our children, the average HbA1c was 10.5%, whereas children with MODY tend to have modest hyperglycemia at diagnosis (HbA1c < 7.5%) [12], and hence, none of them fulfilled this criterion. It is also known that some MODY subtypes have associated clinical features. As an example, children with HNF1B functional variants usually have kidney involvement [8, 26]. The absence of kidney defects in our two children with *HNF1B* rare variants further supports that they do not represent *bona fide* MODY cases. Moving beyond individual cases, we also showed that the overall T1D genetic susceptibility is indistinguishable in our EOT1D children carrying or not MODY gene rare variants, as indicated by a similar T1D-GRS and the presence of high-risk HLA class II haplotypes in all. This contrasts with the reported discriminative power of T1D-GRS in proven MODY [5], where the T1D-GRS of MODY patients is low.

Notably, while in our cohort most clinical features in EOT1D children are similar regardless of the presence of MODY variants, EOT1D children carrying these tend to display at diagnosis a decreased *β*-cell reservoir, despite most presenting a lower extent of islet autoantigen spreading. These findings support the hypothesis of heightened *β*-cell fragility in these participants. MODY genes encode proteins implicated in pancreatic *β*-cell development and function, enzymes involved in the insulin secretion pathway and factors regulating gene expression, including insulin. We therefore propose that the occurrence of deleterious MODY gene variants in children with high T1D genetic burden renders *β*-cells less resistant to autoreactive T-cell attack, thus favoring disease precipitation and clinical EOT1D.

Recent studies suggest that T1D is a heterogenous clinical entity comprising distinct disease subtypes identifiable by unique pancreatic immunophenotypes. Patients diagnosed under 7 years old often present a more aggressive form of insulitis that is characterized by both T- and B-cell infiltrates whereas individuals diagnosed at 13 years or older typically display milder insulitis, primarily characterized by T-cell infiltration [31]. Our findings provide novel insights into T1D heterogeneity by identifying *β*-cell fragility as a new player. These results also provide support to the hypothesis that *β*-cells are not a bystander target of autoreactive T-cells but are instead a key contributor to T1D etiology [14].

According to the International Society for Pediatric and Adolescent Diabetes (ISPAD) guidelines, genetic testing for MODY is recommended in children with diabetes without the classic characteristics of T1D or T2D, with an autosomal dominant family history of diabetes, negative islet autoantibodies, and evidence of preserved *β*-cell function [8]. According to our results, it can be argued that the search for deleterious rare variants in the MODY genes may be considered in a subgroup of T1D children with disease onset before the age of 6 years with no antibodies or low antibody titers. In fact, it is known that children with *HNF1A*, *HNF4A*, *KCNJ11*, and *ABCC8* deleterious mutations are usually well-manageable with sulphonylureas [8, 10, 11]. Children in our cohort with rare MODY variants responsive to sulphonylureas might benefit from a combined therapy of insulin together with an oral agent.

The main strength of this study resides in the cohort of EOT1D children, recruited according to age at onset (T1D diagnosis ≤ 5 years), independently of their immune status at diagnosis, combined with in-depth genetic analysis using WES, multitest *in silico* prediction of functionality, high-resolution HLA class II haplotyping, and SNP genotyping. The reported variants were identified using stringent analysis of WES data, with high coverage, high-quality score and sequencing depth, and high mapping quality at each variant site. Our study also has limitations. The identified rare variants await confirmation by Sanger sequencing. *In silico* prediction for each identified rare variant awaits confirmation in tailored in vitro or ex vivo functional assays. Moreover, we did not scrutinize deleterious MODY rare variants in T1D individuals with later disease onset, which would clarify their role in T1D subtype pathophysiology. Based on our findings, we hypothesize that genetically determined *β*-cell fragility contributes to T1D onset precipitation. It remains to be determined whether this hypothesis is valid in other populations, namely, in patients with different genetic ancestries.

## 5. Conclusions

Children with early diagnosis of T1D may carry functional MODY rare variants, which complete a T1D genetic susceptibility landscape. The synergies of pathophysiological mechanisms involving *β*-cells and immune functions may contribute to T1D endotypes and disease heterogeneity. WES at T1D onset may be a prerequisite for the implementation of precision therapies in children with autoimmune diabetes.

## Figures and Tables

**Table 1:** Potentially deleterious rare variants in MODY genes identified by NGS in the EOT1D cohort.

**Patient**	**Gene**	**Variant (Chr: Pos Ref > Alt)**	**Quality score**	**Sequencing depth**	**Variant classification**	**MAF gnomAD**	**MAF NFSE gnomAD**
1	PDX1	13:28494372 C > G	999	90	Missense	0.0003169	0
2		13:28498518 G > A	793	72	Missense	NP	NP
**3**	**GCK**	**7:44187282 A > ACCG**	**2944**	**163**	**Nonframeshift insertion**	**NP**	**NP**
4	HNF1B	17:36104632 C > T	2841	240	Missense	0.0005831	0.0002589
5		17:36104650 C > A	1696	231	Missense	0.0005500	0.001640
6	HNF4A	20:43052748 A > G	3096	241	Missense	0.000004022	0
7	RFX6	6:117246670 G > C	2163	191	Missense	0.002240	0.005782
8			2766	194			

*Note:* Quality score: Phred-scaled quality score assigned by the variant caller for the assertion made in Alt. Phred scale score = −10 × log_10_(*P*), where *P* is the error probability, and thus, a score of 100 indicates a 1 in 10^10^ chance of error. Sequencing depth: number of unique reads that include the variant site. MAF gnomAD: minor allele frequency retrieved from gnomAD v2.1.1. MAF NFSE gnomAD: minor allele frequency in non-Finnish European Southern Europeans retrieved from gnomAD v2.1.1. The raw highlighted in bold indicates a novel variant (no available documentation in public domain databases).

Abbreviations: Alt, alternate base(s); Chr, chromosome; NP, not present; Pos, reference position; Ref, reference base.

**Table 2:** Computational predictor results for the potentially deleterious rare variants in MODY genes identified in EOT1D participants.

**Missense variants**										
**Patient**	**Gene**	**SIFT**	**PolyPhen-2**	**Mut. Taster**	**CADD**	**Predict SNP**	**M-CAP**	**REVEL**	**Meta LR**	**I-Mutant**
1	PDX1	D	D	D	25.5	D	D	0.718	D	Dec. stab.
2		D	D	A	32.0	D	D	0.958	D	Dec. stab.
4	HNF1B	D	P	D	28.8	D	D	0.890	D	Dec. stab.
5		D	D	D	31.0	D	D	0.915	D	Dec. stab.
6	HNF4A	D	B	D	24.2	N	D	0.713	D	Dec. stab.
7	RFX6	D	D	D	28.2	D	D	0.309	T	Dec. stab.
8										
**Nonframeshift insertions**										
**Patient**	**Gene**	**SIFT Indel**		**Mut. Taster**		**MutPred-Indel**	**VEST-Indel**		**CADD**	
3	GCK	D		D		D	P		22.2	

*Note:* SIFT: D, deleterious. PolyPhen-2: D, probably damaging; P, possibly damaging; B, benign. Mut. Taster (mutation taster): A, disease-causing automatic (i.e., known to be deleterious); D, disease causing (i.e., probably deleterious). Predict SNP: D, deleterious; N, neutral. M-CAP: D, deleterious. MetaLR: D, damaging; T, tolerated. I-Mutant 2.0: Dec. stab., decreased stability. SIFT-Indel: D, deleterious. MutPred-Indel: D, deleterious. VEST-Indel: P, pathogenic. CADD: variants with scores above 20 are predicted to be among the 1.0% most deleterious possible substitutions in the human genome. REVEL: score for an individual missense variant can range from 0 to 1, with higher scores reflecting greater likelihood that the variant is disease-causing. We defined REVEL score threshold ≥ 0.7 for deleterious variants.

**Table 3:** Clinical characterization of EOT1D participants with potentially deleterious rare variants in MODY genes.

**Patient**	**Sex**	**T1D 1st-degree relatives**	**At diagnosis**					**At present**			
			**Age (Y)**	**Presentation**	**HbA1c (%)**	**C-peptide (ng/mL)**	**AutoAb**	**Age (Y)**	**BMI (SD)**	**HbA1c (%)**	**T1DD (IU/kg/day)**
1	M	No	1	DKA	9.2	0.1	GAD	15	−0.1	7.5	1.0
2	F	No	4	Hyperglycemia	13.0	< 0.1	NA	18	1.6	8.2	0.7
3	F	No	4	DKA	12.8	0.2	IA2, IAA	14	1.4	8.0	1.1
4	M	No	2	Hyperglycemia	8.3	< 0.1	IA2	19	0.5	6.8	0.7
5	M	Yes	5	Hyperglycemia	8.4	0.2	No	11	2.2	7.3	0.9
6	M	No	1	DKA	10.4	< 0.1	GAD, IAA	17	0.8	8.6	0.4
7	M	No	3	DKA	10.3	0.1	GAD	11	1.9	7.4	0.9
8	F	No	4	Hyperglycemia	11.3	0.3	GAD	14	2.2	7.2	0.4

Abbreviations: AutoAb, autoantibodies; BMI, body mass index; DKA, diabetic ketoacidosis; F, female; GAD, glutamic acid decarboxylase 65 autoantibodies; HbA1c, glycated hemoglobin; IAA, insulin autoantibodies; IA2, islet antigen 2 autoantibodies; M, male; NA, not available; TIDD, total insulin daily dose; Y, years.

**Table 4:** Comparison of clinical characteristics of EOT1D participants with and without potentially deleterious rare variants in MODY genes.

			**At diagnosis**					
**Group**	**Sex**	**1st-** **degree ** **relatives ** **with T1D**	**Age ** **(** **m** **e** **a** **n** ± **SEM, Y)**	**DKA at ** **presentation**	**HbA1c ** **(** **m** **e** **a** **n** ± **SEM, %)**	**Fasting C-peptide** **(** **m** **e** **a** **n** ± **SEM**, **ng/mL)**	**Presence of ** **AutoAb**	
With MODY variants (*n* = 8)	5/8 male	1/8	3.00 ± 0.54 (*n* = 8)	4/8	10.46 ± 0.64 (*n* = 8)	0.15 ± 0.03 (*n* = 8)	6/7	1–4/6
								≥ 2–2/6
Without MODY variants (*n* = 91)	50/91 male	14/91	2.77 ± 0.16 (*n* = 91)	35/76	10.53 ± 0.20 (*n* = 65)	0.33 ± 0.06 (*n* = 49)	73/75	1–24/73
								≥ 2–49/73

*Note:* The comparison between the two groups yields no statistically significant result for any of the clinical parameters evaluated.

Abbreviations: AutoAb, autoantibodies; DKA, diabetic ketoacidosis; HbA1c, glycated hemoglobin; SEM, standard error of mean; Y, years.

## Data Availability

The datasets presented in this article are not readily available because it contains genotype information that cannot be publish in public repositories. Raw data are available upon a reasonable request via email to the corresponding authors.

## Supporting Information

Supporting Information Additional supporting information can be found online in the Supporting Information section. This included Table S1 with the non-HLA SNPs associated with T1D used to calculate the T1D-GRS, Table S2 with the weight conferred by each HLA class II genotype used to calculate the T1D-GRS, and Table S3 with the HLA class II genotype and T1D-GRS for each EOT1D participant harboring potentially deleterious rare variants in MODY genes.
