# Supplementary material for: Whole Exome Sequencing in Children With Type 1 Diabetes Before Age 6 Years Reveals Insights Into Disease Heterogeneity
Source: J Diabetes Res. 2024 Sep 26;2024:3076895. doi: 10.1155/2024/3076895 (PMC11449554; doi:10.1155/2024/3076895)
Supplement: Supporting Information — Additional supporting information can be found online in the Supporting Information section. This included Table S1 with the non-HLA SNPs associated with T1D used to calculate the T1D-GRS, Table S2 with the weight conferred by each HLA class II genotype used to calculate the T1D-GRS, and Table S3 with the HLA class II genotype and T1D-GRS for each EOT1D participant harboring potentially deleterious rare variants in MODY genes. [file 3076895.f1.docx]

**Table S1:** T1D SNPs included in the genetic score.

| **SNP** | **Gene** | **Odds Ratio** | **Weight** | **Effect allele** |
| --- | --- | --- | --- | --- |
| rs2476601 | PTPN22 | 1.96 | 0.67 | A |
| rs689 | INS | 1.75 | 0.56 | T |
| rs61839660 | IL2RA | 1.43 | 0.36 | C |
| rs2292239 | ERBB3 | 1.35 | 0.30 | T |
| rs10509540 | C10orf59 | 1.33 | 0.29 | T |
| rs4948088 | COBL | 1.3 | 0.26 | C |
| rs7202877 | CTRB1/CTRB2 | 1.28 | 0.25 | G |
| rs127708716 | CLEC16A | 1.23 | 0.21 | A |
| rs3087243 | CTLA4 | 1.22 | 0.20 | G |
| rs1893217 | PTPN2 | 1.2 | 0.18 | G |
| rs7090530 | IL2RA | 1.21 | 0.19 | A |
| rs3024505 | IL10 | 1.19 | 0.17 | G |
| rs9388489 | C6Orf173 | 1.17 | 0.16 | G |
| rs1465788 | MAGOH3P/ZFP36L1 | 1.16 | 0.15 | C |
| rs1990760 | IFIH1 | 1.16 | 0.15 | T |
| rs3825932 | CTSH | 1.16 | 0.15 | C |
| rs425105 | PRKD2 | 1.16 | 0.15 | T |
| rs763361 | CD226 | 1.16 | 0.15 | T |
| rs4788084 | IL27 | 1.16 | 0.15 | C |
| rs11755527 | BACH2 | 1.13 | 0.12 | G |
| rs11203203 | UBASH3A | 1.15 | 0.14 | A |
| rs6827756 | KIAA1109/IL2 | 1.11 | 0.10 | T |
| rs2281808 | SIRPG | 1.11 | 0.10 | C |
| rs5753037 | LIF-AS1/HORMAD2 | 1.11 | 0.10 | T |

Effect allele refers to the risk increasing allele, for which the odds ratio (OR) and corresponding weight (ln(OR)) are indicated.

**Table S2:** Weight conferred by HLA class II genotypes included in the T1D-genetic risk score.

| **HLA class II**  **genotype** | **Odds Ratio** | **Weight** |
| --- | --- | --- |
| DR3/DR4 | 48.18 | 3.87 |
| DR3/DR3 | 21.12 | 3.05 |
| DR4/DR4 | 21.98 | 3.09 |
| DR3/X | 4.53 | 1.51 |
| DR4/X | 7.03 | 1.95 |

DR3: DRB1*03:01-DQA1*05:01-DQB1*02:01 haplotype;

DR4: DRB1*04:01/04:02/04:04/04:05/04:08-DQA1*03-DQB1*03:02 haplotype;

X: another haplotype than DR3 or DR4.

**Table S3:** HLA class II genotype and T1D-GRS in EOT1D participants harboring potentially deleterious rare variants in MODY genes.

| **Patient** | **HLA class II**  **genotype** | **T1D-GRS** | **T1D-GRS classification** |
| --- | --- | --- | --- |
| 1 | DR3/X | 0.177 | High |
| 2 | DR3/DR4 | 0.197 | High |
| 3 | DR4/X | 0.161 | High |
| 4 | DR3/DR3 | 0.187 | High |
| 5 | DR4/X | 0.164 | High |
| 6 | DR3/X | 0.139 | Low |
| 7 | DR3/DR4 | 0.178 | High |
| 8 | DR3/DR3 | 0.189 | High |

DR3: DRB1*03:01-DQA1*05:01-DQB1*02:01 (DR3-DQ2) haplotype;

DR4: DRB1*04:01/04:02/04:04/04:05/04:08-DQA1*03-DQB1*03:02 (DR4-DQ8) haplotype;

X: another haplotype than DR3 or DR4; GRS: Genetic Risk Score.

Parte inferior do formulário
